# Investigating the Readability and Quality of AI Systems to Trending Questions About Food Poisoning

**DOI:** 10.1111/1750-3841.71001

**Published:** 2026-03-28

**Authors:** Idris Demirsoy, Abdullah Dikici

**Affiliations:** ^1^ Department of Computer Engineering, Faculty of Engineering and Natural Sciences Uşak University Uşak Turkey; ^2^ Department of Food Engineering, Faculty of Engineering and Natural Sciences Uşak University Uşak Turkey

**Keywords:** food safety, information quality, large language models, public health, readability

## Abstract

Consumers increasingly turn to artificial intelligence (AI) systems, including search engines and large language models (LLMs), for immediate food safety guidance. However, the reliability and accessibility of this information for critical public health issues, such as food poisoning, remain unassessed. This study benchmarks the performance of major AI systems: Google, ChatGPT, DeepSeek, and Mistral, by simultaneously evaluating the readability and information quality of their responses to frequently asked questions on food poisoning. Readability was assessed using the Flesch–Kincaid Grade Level (FKGL), Simple Measure of Gobbledygook (SMOG), and Gunning‐Fog Index (GFI) indices. Information quality was evaluated by independent experts using the validated DISCERN instrument and Global Quality Scale (GQS). Our analysis revealed a critical divergence in platform performance. Google produced the most readable text (FKGL: 9.05) but the lowest quality information (DISCERN: 30–34; GQS: only 3% of ratings were top‐score). Conversely, LLMs provided high‐quality information (DeepSeek DISCERN: 70–75; ChatGPT: 62) but at significantly higher reading levels (FKGL: 10.01–11.32), exceeding the recommended sixth‐grade level. This demonstrates a fundamental trade‐off: search engines optimize for brevity and accessibility, whereas dedicated LLMs prioritize comprehensive, reliable content. This forces consumers to choose between understandable but potentially misleading information and accurate but inaccessible guidance. Our findings highlight an urgent need to bridge this gap between readability and quality, calling for the development of AI systems that deliver authoritative, comprehensible food safety advice to protect public health.

**Abbreviations**
AI: artificial intelligence
ANOVA: analysis of variance
ERISS: Emerging Risk Identification and Screening System
FKGL: Flesch–Kincaid Grade Level
FSAI: Food Safety Authority of Ireland
GFI: Gunning‐Fog Index
GQS: Global Quality Scale
ICC: intraclass correlation coefficient
LLM: large language models
Max: maximum
Min: minimum
NTU: Nanyang Technological University
SD: standard deviation
SFA: Singapore Food Agency
SMOG: the Simple Measure of Gobbledygook

## 1 Introduction

Consuming unsafe foods can lead to foodborne illnesses caused by bacteria, viruses, parasites, chemical, or physical agents. Food poisoning can cause acute or chronic symptoms, ranging from diarrhea to cancer, permanent disability, and death, and can lead to nearly 200 different diseases (WHO 2022). Every year, 600 million people worldwide, or roughly one in 10 people, fall ill due to food poisoning. Globally, foodborne diseases account for an estimated 420,000 deaths and 33 million disability‐adjusted life years (DALYs) lost each year, reflecting a significant public health burden (WHO 2015). This impact is unevenly distributed, with developing countries bearing a disproportionate share of both morbidity and mortality, alongside an estimated global economic loss of $110 billion per year attributable to food poisoning (WHO 2022).

Concurrently, the digital revolution has transformed public access to information. Individuals increasingly turn to online sources by asking questions on different search engines to get ideas about their potential problems (Diaz et al. 2002; M. Li et al. 2019; Urtekin and Kartal 2025; Özbek et al. 2025). In 2018, nine out of ten American adults used the World Wide Web, and 75% of them conducted medical searches (Gunduz et al. 2024). Artificial intelligence (AI) technologies, including large language models (LLMs), are revolutionizing the accessibility and dissemination of health information. A growing number of medical queries, including those concerning food poisoning symptoms, are now directed to AI methods (Lawson McLean 2024). Commonly used systems such as ChatGPT, DeepSeek, Gemini, and the AI‐enhanced Google Search engine are now frequently consulted by the public for health information. We collectively refer to these methods as AI systems in this study.

Meanwhile, AI applications are rapidly developing in areas that directly affect public health, such as food safety. In the early stages of AI implementation in food safety, the primary focus was on automating data analysis and improving decision‐making processes (Huang et al. 2019). The Singapore Food Agency (SFA), in collaboration with Nanyang Technological University (NTU), has developed a system that uses language models to retrieve, categorize, and analyze news articles on food safety from online sources. SFA and NTU have also created an automated system that uses LLMs to scan scientific literature and filter publications on foodborne pathogens (van Meer et al. 2025). In addition, the Food Safety Authority of Ireland (FSAI) uses LLMs as part of its Emerging Risk Identification and Screening System (ERISS) (van Meer et al. 2025). Researchers examined how AI and machine learning have transformed the detection and response to food poisoning outbreaks, noting that AI models can process large amounts of health‐related data in real time, enabling faster interventions than traditional methods (Maroney 2025). A scientist reports that a researcher from Southern Illinois University has developed a rapid AI‐based method to detect pathogens such as *Salmonella*, which cause food poisoning, before they enter the food supply chain. Mitchell (2025) discussed leveraging AI in food safety, noting that AI has become a fundamental tool for enhancing human capabilities and enabling smarter, faster decisions across the industry. Friedlander and Zoellner (2020) examined AI opportunities to improve food safety in the retail sector, highlighting AI's potential to advance and transform food safety practices and outcomes.

The use of AI‐driven language models has increased substantially for accessing medical information and supporting patient education (Collins et al. 2025; Ozduran et al. 2025; Sahin et al. 2025). Their popularity stems from their ability to provide rapid responses, particularly for health‐related questions. However, realizing the potential of AI‐based chatbots to simplify and make health information more accessible requires careful evaluation. The National Institutes of Health, the US Department of Health and Human Services, and the American Medical Association recommend that patient education materials should be written to a Grade 6 level (Ozduran et al. 2024). For example, researchers examined the readability of responses from AI chatbots regarding back pain. They noted that these responses were above the recommended readability level (sixth‐grade) (Ozduran et al. 2025), complicating comprehension for the average reader. Similarly, Collins et al. (2025) found that although Gemini 1.0 offered better readability in responses about Achilles tendon injuries, quality varied significantly across models (Collins et al. 2025). Conversely, researchers concluded that educational content generated by ChatGPT‐3.5 was faster to read, easier to understand, and rated higher in quality than text written by humans (Rashid et al. 2024). Together, these contrasting findings highlight that readability and information quality are critical, interdependent factors determining the real‐world utility of AI‐generated health advice.

Despite AI systems’ growing role in digital health communication and food safety systems, the accuracy, reliability, and clarity of the consumer health information they provide on common yet serious topics, such as food poisoning, have not been comprehensively evaluated. Given the widespread nature of foodborne illnesses and the public's increasing reliance on AI tools for health guidance, it is essential to assess the quality of the information these models deliver in this area (Abbasian et al. 2024). For this study, we selected well‐known AI systems, including ChatGPT (OpenAI, USA), DeepSeek (China), Mistral (France), and the AI‐supported search engine Google (Google, USA), based on their popularity among users and regional relevance. Although the readability of AI applications has been evaluated in many clinical areas, to the best of our knowledge, no study has specifically examined food poisoning information. Thus, this study aims to conduct a pioneering evaluation of the readability and quality of answers provided by different AI systems to frequently asked questions about food poisoning.

## 2 Methods

This cross‐sectional study was conducted in accordance with the principles outlined in the Declaration of Helsinki. This research involved only automated analysis of machine‐generated text outputs from publicly accessible AI systems (Google, ChatGPT, DeepSeek, and Mistral), using standard readability formulas (Flesch–Kincaid, Gunning‐Fog Index [GFI], and Simple Measure of Gobbledygook [SMOG]) on non‐sensitive data. Institutional review board approval was not applicable.

To establish a clinically relevant and ecologically valid set of questions, we identified the 15 most frequently searched queries related to “food poisoning” using Google Trends (data extracted on February 3, 2025). This approach ensured our analysis reflected genuine public information‐seeking behavior. The selected questions are listed in Table S1.

### 2.1 Data Collection

We selected four platforms based on global popularity, regional representation, and relevance to public health information‐seeking behavior. ChatGPT (OpenAI, USA; version GPT‐4o), DeepSeek (DeepSeek, China; version V2), and Mistral (Le Chat AI, France; version Mistral Large) were chosen as leading LLMs from key technological regions, ensuring architectural and developmental diversity. Google was included not as a standalone LLM (e.g., Gemini), but as the dominant search engine whose AI‐generated “Overview” snippets serve as the primary source of instant health information for most users globally (because Google controls approximately 92% of global search traffic). This selection enables a comparative analysis of dedicated LLM chatbots and the AI‐augmented search results most frequently encountered by the public. All questions were entered in English via a web browser (Chrome Version 140.0.7339.132) without any additional prompting or context to simulate a typical user's interaction. The free, publicly accessible versions of all AI models were used through their official websites. After each query, caches and cookies were cleared to avoid bias.

### 2.2 Readability Assessment Methods

Readability was evaluated using three validated indices: the Flesch–Kincaid Grade Level (FKGL), SMOG, and GFI Index. These were selected for their widespread use and validation in health communication research. The indices quantify the US academic grade level required to comprehend a text based on structural features like sentence length and syllable count (Kara et al. 2025; Pradhan et al. 2024). Detailed formulas and validation criteria are provided in Supporting Information S1. All analyses were conducted using an open‐access, web‐based readability calculator (https://readabilityformulas.com) to ensure consistency (Kher et al. 2017).

### 2.3 Quality Assessment

The Global Quality Scale (GQS) offers a structured framework for assessing the overall methodological soundness and practical utility of information sources. Instead of focusing on isolated metrics, it evaluates the study's comprehensive reliability and validity. This scale assigns a score from 1 to 5 based on the organization and depth of content, with lower scores indicating high bias, minimal information sources, and the highest score reserved for comprehensively covered, excellently structured research (Table S2) (Bernard et al. 2007). This approach allows for an assessment of overall validity rather than focusing on specific metrics (Eroglu and Altinli 2022).

To systematically evaluate the quality of health information, we employed the DISCERN tool. This validated questionnaire, developed at Oxford University, comprises 16 questions segmented into three domains: publication reliability, specifics of treatment options, and an overall rating. Each item is scored on a 5‐point scale, facilitating a systematic assessment of a source's credibility, potential bias, and practical utility for clinical or patient decision‐making (Table S3) (Boyer et al. 1998). The DISCERN and GQS tools evaluate the clarity, balance, transparency, and scope of health information but do not verify its scientific or medical accuracy.

### 2.4 Statistical Analysis

The numerical data were described using means and standard deviations. Normality check was assessed using the Shapiro–Wilk test. The accuracy and quality of responses generated by AI systems were evaluated by two independent, blinded experts (TA, SBB), who evaluated all responses. Both raters underwent a calibration session using sample texts not included in the study to ensure consistent application to use DISCERN and the GQS. Inter‐rater reliability for the GQS scores was assessed using Cohen's kappa coefficient (Demirsoy et al. 2025) based on a two‐way random‐effects model. DISCERN total scores were evaluated using the intraclass correlation coefficient (ICC). The analysis indicated substantial agreement between the two expert evaluators. Differences in AI system performance across readability indices were analyzed with a two‐way repeated‐measures ANOVA, followed by post hoc pairwise comparisons with Bonferroni corrections. The generalized eta‐squared (GES) was used to report effect sizes. All statistical analyses were carried out using R programming software (version 4.4.1) (R Core Team 2023).

## 3 Result

**FIGURE 1:**
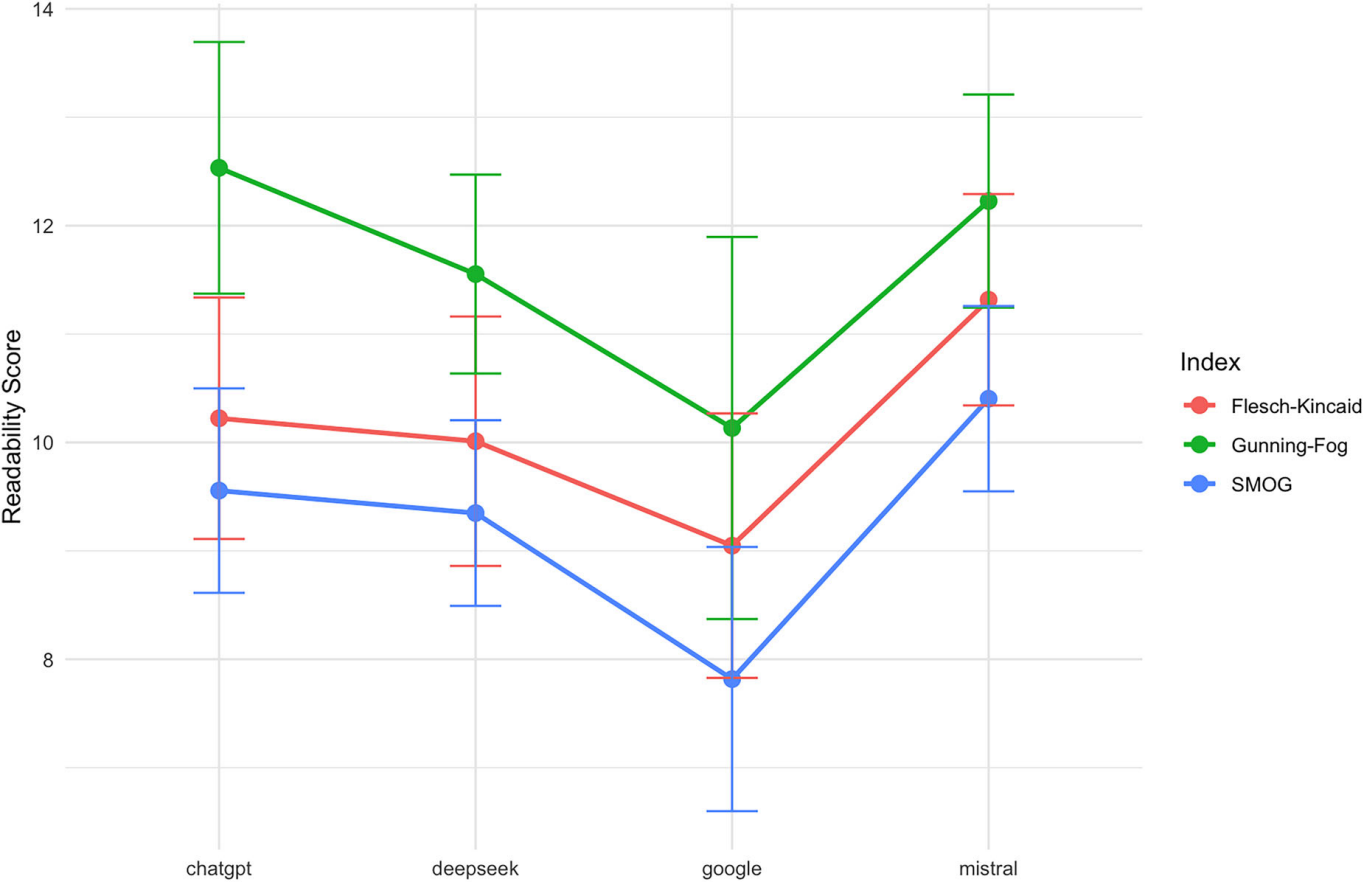
Interaction term with method and readability grade level indexes. SMOG, Simple Measure of Gobbledygook.

Analysis of readability indices revealed a clear hierarchy of response complexity across the four platforms (Table 1). Google consistently generated the most accessible content, achieving the lowest mean grade level across all three indices, including the lowest mean SMOG index of 7.82. Google showed the most significant variability in scores, particularly on the GFI index (SD = 3.18), suggesting less predictable readability across questions (Figure 1). In contrast, ChatGPT, DeepSeek, and Mistral produced text of notably higher complexity, with nearly all mean scores exceeding the eighth‐grade level. Mistral consistently required the highest grade level for comprehension and demonstrated the most consistent performance, with the smallest standard deviation across all three indices (SD = 1.54).

**TABLE 1:** Descriptive statistics of readability grade levels by method and index.

Method	Index	Mean	SD	Min	Max
ChatGPT	FKGL	10.22	2.01	6.48	14.02
	GFI	12.53	2.10	9.40	16.90
	SMOG	9.56	1.70	7.20	13.16
DeepSeek	FKGL	10.01	2.08	6.17	13.27
	GFI	11.55	1.66	8.40	14.40
	SMOG	9.35	1.55	6.99	12.26
Google	FKGL	9.05	2.20	4.85	12.07
	GFI	10.13	3.18	3.50	16.00
	SMOG	7.82	2.20	3.57	11.09
Mistral	FKGL	11.32	1.76	7.88	13.48
	GFI	12.23	1.77	8.40	14.00
	SMOG	10.40	1.54	6.91	12.39

Abbreviations: FKGL, Flesch–Kincaid Grade Level; GFI, Gunning‐Fog Index; Max, maximum; Min, minimum; SD, standard deviation; SMOG, Simple Measure of Gobbledygook.

### 3.1 Multiple Comparison

The two‐way repeated measures ANOVA revealed significant and large main effects of methods (*p* = 0.001, *η* ^2^ *G* = 0.161) and a substantial main effect of readability index (*p* < 0.001, *η* ^2^ *G* = 0.194), along with a small but significant interaction between method and readability index (*p* = 0.011, *η* ^2^ *G* = 0.015).

There is noticeable variability in readability scores across different questions for each method and readability index. This suggests that the readability of answers can vary significantly depending on the question. The method and index effects are significant, indicating differences in readability scores across methods and indices.

Post‐hoc pairwise comparisons revealed a clear performance hierarchy (Table 2). Google consistently generated more readable text than other AI systems, especially showing significant advantages over Mistral across all three indices (FKGL: *p* = 0.0016; GFI: *p* = 0.0042; SMOG: *p* = 0.0003) and over ChatGPT on both GFI (*p* = 0.0008) and SMOG (*p* = 0.0254) indices. In contrast, the three dedicated AI models, ChatGPT, DeepSeek, and Mistral, formed a distinct cluster, with no statistically significant differences in readability among them across any index. The consistently low estimates and nonsignificant *p* values (all >0.05) indicate their outputs were closely matched in complexity, despite Mistral trending toward the highest reading demands. All reported *p* values were adjusted using the Bonferroni method for multiple comparisons.

**TABLE 2:** Pairwise comparisons of readability grade levels by index.

Index	Comparison	Estimate	*t*‐ratio	*p* value
FKGL	ChatGPT–DeepSeek	0.212	0.363	1.0000
FKGL	ChatGPT–Google	1.175	2.014	0.2924
FKGL	ChatGPT–Mistral	−1.093	−1.873	0.3968
FKGL	DeepSeek–Google	0.963	1.650	0.6258
FKGL	DeepSeek–Mistral	−1.305	−2.236	0.1753
FKGL	Google–Mistral	−2.269	−3.887	0.0016^*^
GFI	ChatGPT–DeepSeek	0.980	1.679	0.5915
GFI	ChatGPT–Google	2.400	4.112	0.0008^*^
GFI	ChatGPT–Mistral	0.307	0.525	1.0000
GFI	DeepSeek–Google	1.420	2.433	0.1087
GFI	DeepSeek–Mistral	−0.673	−1.154	1.0000
GFI	Google–Mistral	−2.093	−3.586	0.0042^*^
SMOG	ChatGPT–DeepSeek	0.207	0.355	1.0000
SMOG	ChatGPT–Google	1.739	2.979	0.0254^*^
SMOG	ChatGPT–Mistral	−0.847	−1.452	0.9121
SMOG	DeepSeek–Google	1.531	2.624	0.0667
SMOG	DeepSeek–Mistral	−1.055	−1.807	0.4560
SMOG	Google–Mistral	−2.586	−4.431	0.0003^*^

*Note*: All *p* values are Bonferroni‐adjusted for multiple comparisons. SE = 0.584 for all comparisons.

Abbreviations: FKGL, Flesch–Kincaid Grade Level; GFI, Gunning‐Fog Index; SMOG, Simple Measure of Gobbledygook.

^*^p < 0.05

### 3.2 Item‐Level Analysis

**FIGURE 2:**
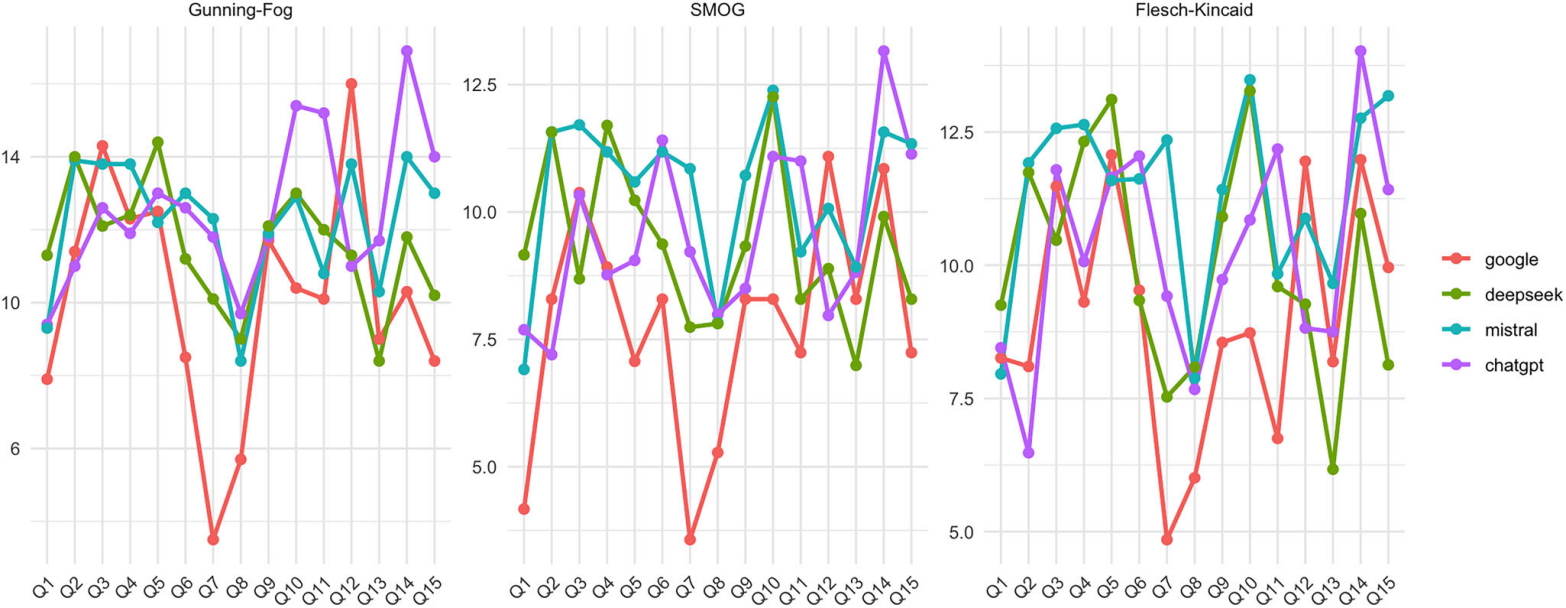
Readability scores of answers generated by different methods (Google, DeepSeek, Mistral, ChatGPT) across various questions, evaluated by three readability indexes (GFI, SMOG, FKGL). Each subplot represents a different readability index. SMOG, Simple Measure of Gobbledygook.

For the GFI index, readability scores fluctuate across questions for each method, with Google showing notable dips around the questions “What should we do in case of food poisoning?” (Q7) and “How do you tell if you have Salmonella?” (Q12), whereas DeepSeek, Mistral, and ChatGPT maintain relatively more consistent scores with some fluctuations. Similar patterns are observed for the SMOG index, where Google's scores again dip significantly. *What are the symptoms of food poisoning? (*Q1), Q7, Q12, and *Why is food poisoning so painful? (*Q14), and the other methods exhibit more stable scores with some variations. The FKGL index also reveals fluctuations in readability scores across questions for each technique, with Google's scores dipping on Q7. At the same time, DeepSeek, Mistral, and ChatGPT display more consistent scores with some fluctuations. Overall, independent of the readability indexes, Google shows that Q7 is the lowest grade question to read, whereas Q12 is the hardest grade question to read (Figure 2).

### 3.3 Inter‐Rater Reliability and Quality Assessment

Evaluation using the GQS revealed a pronounced contrast in information quality. DeepSeek and ChatGPT provided the highest‐quality responses, with DeepSeek achieving the maximum score of 5 in 70% of ratings (21/30) and ChatGPT in 63.33% (19/30). In contrast, Google's responses were consistently rated as the lowest quality, with only 3.33% of ratings (1/30) reaching the highest score (5) and 30% of ratings (9/30) falling into the lowest quality categories (scores of 1 or 2). This poor performance was consistent between raters (*κ* = 0.734), with both experts assigning the lowest possible score (1) to two specific questions: *What's the difference between food poisoning and the stomach bug?* (Q9), and *what color is diarrhea with food poisoning?* (Q10).

**FIGURE 3:**
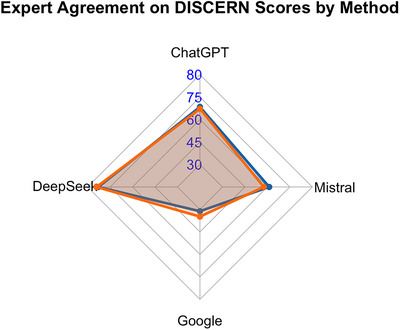
DISCERN scores for methods versus experts.

The item‐level analysis using the DISCERN instrument revealed a different pattern from that observed with the GQS. The agreement, as measured by Cohen's kappa, was almost perfect for DeepSeek (*κ* = 0.803, *p* = 0.001) and ChatGPT (*κ* = 0.885, *p* < 0.001), and substantial for Mistral (*κ* = 0.775, *p* = 0.001) and Google (*κ* = 0.714, p = 0.002). This high level of concordance underscores the consistency of the expert evaluations. However, this agreement also highlights the poor quality of Google's outputs (Figure 3). Despite consistent scoring, the experts assigned Google very low total DISCERN scores of 30 and 34, indicating that the information was seriously lacking in quality and reliability. In contrast, the LLMs, particularly DeepSeek (scores of 74 and 75) and ChatGPT (scores of 62 and 61), not only achieved high inter‐rater agreement but were also consistently rated as providing good‐quality information.

## 4 Discussion

As digital transformation is increasingly embraced across many sectors, particularly in healthcare, AI has emerged as a powerful tool for providing consumers with health information. From chatbots that answer medical questions to AI‐generated patient education materials, these technologies promise to bridge the gap between complex medical information and consumers' levels of understanding. With the rapid development of AI and AI‐backed search engines, individuals with varying levels of education, income, and social status may be able to access equal services and information. However, there are concerns about whether the information obtained is sufficiently readable and understandable for all segments of society. This study directly compared the readability and comprehensibility of information on “food poisoning” obtained from leading AI‐powered platforms and a search engine. Our analysis revealed a critical trade‐off: Search engines like Google, optimized for brevity and readability, often provide lower quality information, whereas dedicated LLMs prioritize comprehensive, high‐quality content at the expense of accessibility. This dichotomy forces consumers to choose between understandable but potentially misleading information and accurate but inaccessible guidance, presenting a significant challenge for public health communication.

### 4.1 Implications for Theory

The methodological framework of our study employed three established readability indices (FKGL, GFI, SMOG), which provide limited assessments as they rely on quantitative features and cannot capture qualitative aspects of comprehension. To complement these, we used the GQS and the DISCERN tool, which evaluate clarity, balance, transparency, and scope of health information. Our findings contribute to theoretical understanding in several ways.

First, the observed inverse relationship between readability and information quality supports the information richness–accessibility trade‐off hypothesis in digital health communication. This trade‐off is structurally embedded in platform design: search engines retrieve and rank existing web pages, resulting in heterogeneous content that can vary significantly in depth and reliability, particularly for specific questions. In contrast, LLMs synthesize information learned from various sources to produce comprehensive, fluent, and structurally consistent responses. This explains why LLM‐generated responses tend to perform better in the scope, consistency, and clarity domains, as evaluated by both the DISCERN instrument and the GQS.

Second, our findings align with and contrast with emerging literature. For instance, although Bahçeci et al. (2025) found Copilot more readable than Google on infertility topics, and Li et al. (2025) noted both Google and ChatGPT produced high‐complexity text on systemic lupus erythematosus, our study reveals the critical trade‐off between readability and quality that refines this understanding (Bahçeci et al. 2025; K. Li et al. 2025). This suggests that theoretical models of AI‐health communication must account for platform‐specific design priorities and their impact on information delivery.

### 4.2 Implications for Practice and Policy

In our study, it was observed that although traditional search engines such as Google Search produced lower GQS scores for specific questions on the topic of “food poisoning” (What's the difference between food poisoning and the stomach bug?, What color is diarrhea with food poisoning?), LLMs such as ChatGPT, Mistral, and DeepSeek achieved higher quality scores. A similar situation was found in DISCERN (Figure 3). This difference stems from the two systems' ways of processing information. Search engines retrieve and rank existing web pages; these pages can vary significantly in terms of content depth, language structure, and reliability. This heterogeneity particularly lowers quality scores for specific questions. In contrast, LLMs synthesise information learned from various sources to produce comprehensive, fluent, and structurally consistent responses (van Meer et al. 2025). As a result, LLM‐generated responses tend to perform better in the scope of information, consistency, and clarity domains evaluated by both the DISCERN instrument and the GQS (Ayers et al. 2023; Sahin et al. 2025; Shiferaw et al. 2024; Sun et al. 2024; Urtekin and Kartal 2025). Although no studies have yet examined AI‐generated information on food poisoning, findings from related health domains indicate that LLMs generally produce higher quality content and greater narrative coherence than traditional web‐based sources (Shiferaw et al. 2024; Sun et al. 2024).

Even if our study identified that LLMs and Google Search results require a higher level of education, LLMs themselves hold the key to mitigating this issue. Evidence shows that LLMs can be strategically prompted to simplify complex medical information effectively. For instance, when tasked with improving the readability of content from major health organizations, LLMs significantly reduced the reading grade level. For example, ChatGPT's output improved from 10.1 to 7.6 (Will et al. 2025). This aligns with findings that targeted prompting can systematically enhance the accessibility of LLM‐generated responses (Musheyev et al. 2024). Considering that at least 40 million adults in the United States have literacy skills below the fifth‐grade level (Kirsch 1994), a similar situation may exist worldwide. Using LLMs to tailor information clarity becomes a necessary step for public health. By doing so, we can leverage these tools to advance health equity, ensuring vital medical information is understandable for everyone. Even if LLMs produced hard‐to‐read texts, when given explicit instructions to respond at a sixth‐grade reading level, LLMs' terminology density decreased, resulting in a significant improvement in the text's readability (Mohammadi et al. 2025; Shen et al. 2024).

### 4.3 Limitations of the Study and Future Research Directions

Several limitations of this study should be considered. First, the analysis was based on a sample of 15 questions, drawn from the most frequent Google Searches to ensure real‐world relevance. However, these 15 questions do not encompass all possible subtopics of food poisoning. The result depends heavily on the selected question set. Future studies with greater resources could expand the query set. Furthermore, our study is subject to sampling bias (English‐language, popular queries) and responses due to the constraints of the readability indices employed from a single web‐based calculator. Consequently, our findings may not be generalizable to other linguistic contexts. Third, we evaluated only four methods, selected for their global popularity and regional diversity to ensure ecological validity. However, the rapidly expanding ecosystem of AI systems means many other models were omitted. Fourth, to maintain a fair comparison and simulate a typical user's experience, we used the default, free versions of these systems without specialized prompts to enhance readability or quality. This choice reflects real‐world usage but does not test the methods' full potential. According to a study, paying for LLMs yields more easily readable responses, but there is no change in the quality of the information (Musheyev et al. 2024). Fifth, the methodological framework for evaluating AI outputs on food poisoning was developed for this pioneering study, drawing on established principles from adjacent fields in the absence of a direct precedent. Sixth, our findings are constrained by the inherent limitations of standard readability formulas (FKGL, GFI, SMOG), which quantify structural text features but cannot assess conceptual complexity, navigational ease, or a user's actual comprehension. Finally, this study provides a static snapshot of dynamic technologies. The performance of both LLMs and search engine algorithms is subject to continuous change through updates. Therefore, temporal instability is expected as AI models and search algorithms evolve rapidly. Replication at future time points is needed to assess consistency. Similarly, the use of free versions, which may have more limited reasoning depth and access to current information than their paid counterparts, represents a snapshot of a specific service tier. Despite these limitations, which are both acknowledged and addressable, this study provides a critical foundational assessment of AI‐generated consumer health information on food poisoning.

## 5 Conclusion

In conclusion, our analysis reveals a critical challenge in digital health communication: The information on food poisoning provided by major AI systems, including Google, is written at a reading level that exceeds public health recommendations and the literacy skills of a significant portion of the population. This study identifies a pivotal trade‐off: The superior readability of search engine outputs often comes at the expense of information quality and comprehensiveness, a gap that is filled by more complex yet higher quality responses from dedicated LLMs. The path forward is clear. Developers and public health authorities must prioritize the intentional design of these tools to bridge this gap. By implementing optimized prompting strategies to generate responses at a sixth‐grade level or below without sacrificing factual accuracy or depth, we can harness the power of AI to democratize health information truly. Such an effort is not merely a technical improvement but a necessary step toward achieving health equity, ensuring that everyone, regardless of literacy level, has access to understandable, trustworthy guidance on critical health issues such as food poisoning.

## Author Contributions

**Idris Demirsoy**: conceptualization, investigation, writing – original draft, methodology, validation, visualization, data curation. **Abdullah Dikici**: methodology, validation, conceptualization, investigation, writing – original draft, supervision, project administration, data curation.

## Conflicts of Interest

The authors declare no conflicts of interest.

## Supporting information




**Supplementary Material**: jfds71001‐supp‐0001‐SuppMat.docx

## Data Availability

The data supporting the findings of this study consist of textual responses generated by the publicly accessible AI systems ChatGPT, DeepSeek, and Mistral, as well as the Google Search engine. These data were generated by the authors using the 15 questions listed in Table S1 and are available in the Supplementary Data files. Due to the dynamic nature of these AI systems, identical responses cannot be guaranteed upon replication. Readability analysis was conducted using the open‐access, web‐based Readability Formulas platform (https://readabilityformulas.com). Statistical analyses were performed using R (version 4.4.1) with standard packages, and the code can be provided upon request.
