# Supplementary material for: Investigating the Readability and Quality of AI Systems to Trending Questions About Food Poisoning
Source: J Food Sci. 2026 Mar 28;91(4):e71001. doi: 10.1111/1750-3841.71001 (PMC13032161; doi:10.1111/1750-3841.71001)
Supplement: Supplementary file 1 — Supplementary Material: jfds71001‐supp‐0001‐SuppMat.docx [file JFDS-91-0-s001.docx]

Supplementary

Table S1

| Qs | Questions |
| --- | --- |
| Q1 | What are the symptoms of food poisoning? |
| Q2 | How long does food poisoning last? |
| Q3 | What is food poisoning? |
| Q4 | How is food poisoning treated? |
| Q5 | What are the microbes that cause food poisoning? |
| Q6 | How is food poisoning transmitted? |
| Q7 | What should we do in case of food poisoning? |
| Q8 | How can I prevent food poisoning? |
| Q9 | What's the difference between food poisoning and the stomach bug? |
| Q10 | What color is diarrhea with food poisoning? |
| Q11 | Do you always vomit with food poisoning? |
| Q12 | How do you tell if you have Salmonella? |
| Q13 | What to eat in food poisoning? |
| Q14 | Why is food poisoning so painful? |
| Q15 | Does chugging water help food poisoning? |

Table S2 Global Quality Scale (GQS) Assessment Criteria

| Score | Quality | Information Content |
| --- | --- | --- |
| 1 | Low Quality | Poor quality, flow, and very limited information |
| 2 | Below Average Quality | Limited information given, but most of them missing |
| 3 | Moderate Quality | Little informing, average useful |
| 4 | High Quality | Well-structured, good quality, flow, and useful. |
| 5 | Excellent quality | Excellent organization, very useful |

Table S3 DISCERN evaluation criteria

| Publication Reliability |
| --- |
| 1. Are the aims clear? |
| 2. Does it achieve its aims? |
| 3. Is it relevant? |
| 4. Is it clear what sources were used? |
| 5. Is it clear when information was produced? |
| 6. Is it balanced and unbiased? |
| 7. Does it provide additional support? |
| 8. Does it refer to areas of uncertainty? |
| 9. Does it describe treatment benefits? |
| 10. Does it describe treatment risks? |
| 11. Does it describe what would happen without treatment? |
| 12. Does it describe the effects on quality of life? |
| 13. Is choice of treatment made clear? |
| 14. Does it provide support for shared decision-making? |
| 15. Does it describe how treatments affect symptoms? |
| 16. Based on answers to all questions, rate overall quality |

**Readability Assessment Methods**

1. **Flesch-Kincaid Grade Level (FKGL)**

One of the most commonly used tools for measuring readability is the Flesch-Kincaid Grade Level (FKGL), which is derived from the Flesch Reading Ease (FRE) formula. It calculates the reading grade level based on the average number of words per sentence and syllables per word. The final score reflects the grade level typically required to comprehend the text. A lower score indicates material that is easier to read, while a higher score suggests the material is more complex and requires a higher level of education to understand. FKGL is commonly applied in education, government communications, and healthcare to ensure textual clarity^1,2^.

$$FKGL = (0.39 \times[total words/total sentences]) + (11.8 \times[total syllables/total words]) - 15.59$$

1. **Gunning Fog Index (GFI)**

The Gunning Fog Index (GFI) estimates the years of formal education level a reader needs to understand a text upon first reading^3^. It assesses text difficulty by analyzing average sentence length and the proportion of complex words, specifically, those with three or more syllables. A higher GFI score indicates increased complexity, suggesting that the content requires a higher level of education to understand. This readability formula is particularly useful for evaluating business, technical, and academic writing.

$$GFI = 0.4\times\left( \left[ {Words}/{Sentences} \right]+100\times\left[ {Complex Words}/{Words} \right] \right)$$

1. **Simple Measure of Gobbledygook (SMOG) Index**

The Simplified Measure of Gobbledygook (SMOG) index, developed by McLaughlin (1969), quantifies readability by estimating the required U.S. grade level for comprehension. Unlike other formulas, SMOG specifically uses the count of polysyllabic words (words with three or more syllables) and the number of sentences to calculate the readability grade level^4^. It is regarded as particularly dependable for evaluating the readability of short medical documents^5^. A higher score of SMOG suggests that the text is more complex and requires a higher level of education to understand^6^.

$$SMOG=1.0430*\sqrt{\left( Polysyllabic Words \times\left( {30}/{Sentence Count} \right) \right)}+3.1291$$

The three readability measures; FKGL, GFI, and SMOG, quantify the U.S. academic grade level (years of formal education) required to understand a given text. For example, a FKGL score of 5.3 indicates fifth-grade readability, a GFI of 12 requires high-school comprehension, and a SMOG score of 13 suggests college-level understanding. To minimize computational inaccuracies and streamline analysis, AI-generated content was assessed for readability using an open-access, web-based readability evaluation tool (https://readabilityformulas.com/readability-scoring-system.php)^7^.

1. Kincaid JP, Fishburne Jr RP, Rogers RL, Chissom BS. Derivation of new readability formulas (automated readability index, fog count and flesch reading ease formula) for navy enlisted personnel. 1975;

2. Flesch R. A new readability yardstick. *Journal of applied psychology*. 1948;32(3):221.

3. Gunning R. The technique of clear writing. *(No Title)*. 1952;

4. Mc Laughlin GH. SMOG grading-a new readability formula. *Journal of reading*. 1969;12(8):639-646.

5. Jido JT, Al-Wizni A, Le Aung S. Readability of AI-Generated Patient Information Leaflets on Alzheimer’s, Vascular Dementia, and Delirium. *Cureus*. 2025;17(6)

6. Pradhan F, Fiedler A, Samson K, Olivera-Martinez M, Manatsathit W, Peeraphatdit T. Artificial intelligence compared with human-derived patient educational materials on cirrhosis. *Hepatol Commun*. Mar 01 2024;8(3)doi:10.1097/HC9.0000000000000367

7. Kher A, Johnson S, Griffith R. Readability Assessment of Online Patient Education Material on Congestive Heart Failure. *Adv Prev Med*. 2017;2017:9780317. doi:10.1155/2017/9780317
